# Supplementary material for: A tipping point in stable isotope composition of Antarctic meteoric waters during Cenozoic glaciation
Source: Nat Commun. 2024 May 27;15:4509. doi: 10.1038/s41467-024-48811-2 (PMC11530553; doi:10.1038/s41467-024-48811-2)
Supplement: Supplementary file 1 — Supplementary Information [file 41467_2024_48811_MOESM1_ESM.pdf]

Supplementary Information: Data & Tables

Samples

The Tertiary intrusive bodies were collectively grouped as the Meander Intrusives unit in the geological map drawn by the GANOVEX Team (1987). The lithotypes in the present study are diorites, monzonite and syenite, respectively, which represent the most abundant rocks of this unit, and crop out roughly parallel to the Ross Sea coast, between the Campbell and Borchgrevink glaciers (Fig. 1_SI). Minor gabbros and diffuse felsic and mafic dykes also occur. In this study, the geochronological framework is based on the $^{40}Ar/^{39}Ar$ from (Dallai & Burgess, 2011). Additional information on samples and coordinates can be found at: https://mna.it/collezioni/catalogo-rocce-sede-di-siena. Rock samples are available at the Rock Repository of the National Antarctic Museum in Siena (IT).

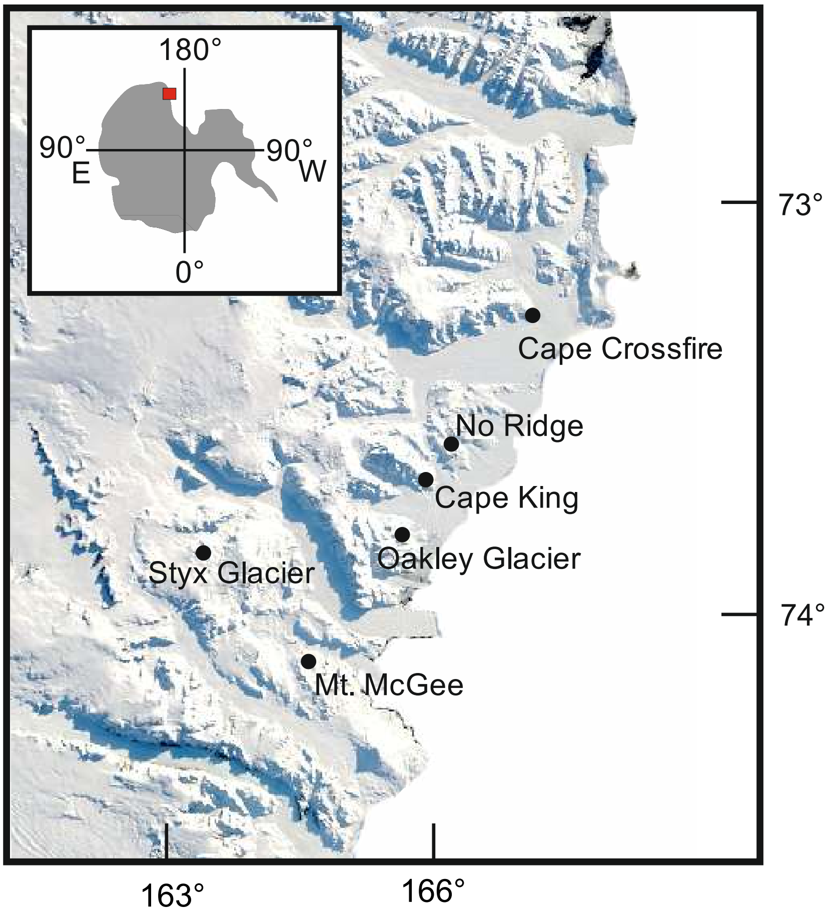

Fig. 1_SI Geological sketch map of the Ross Sea coastline in northern Victoria Land, Antarctica, and sampling sites of the studied intrusive rocks. The investigated region is represented by the little red square in the inset.

Styx Glacier

Monzonite and syenite samples were samples in scattered outcrops within the glacier. They show a diffuse alteration, mainly affecting Kfeldspar. They show a medium-grained to large-grained texture, characterized by the occurrence of abundant k-feldspar and quartz, subordinate zoned albite bytonite plagioclase, and minor biotite, hornblende and opaques (mainly Magnetite and, ilmenite).

Mount McGee

A NW-SE elongated monzonite-syenite body, showing large-grained texture characterized by K-feldspar, quartz, and subordinate amphibole and biotite, intruded the crystalline basement formed. The basement is formed by Cambro Ordovician peraluminous granites. Their mineral assemblage consists of quartz and K-feldspar, subordinate plagioclase, biotite, and minor muscovite. Hydrothermal alteration is confined to the margins of Cenozoic intrusion.

(South) Oakley Glacier

Between the Southern flank of Oackley Glacier and the NE crest of Mt Knetch a monzonite-syenite crops out alternate with coeval diorite intrusions. It shows a medium-grained texture, characterized by the occurrence of Kfs, Qtz, Mg-Fe-Mn-amphibole, rare orthopiroxene, and apatite. Accessory phases are allanite and ilmenite.

Cape King

A dioritic intrusioncrops out on the cliff where a weather antenna is located. The mineral assemblage consists of andesitic plagiocalse, minor quartz, honeblenditic amphibole. Accessory phases are titanite and, magnetite.

Cape Crossfire

Monzonic outcrops occur along the steep cliffs and along the SE ridge of the unnamed mountain north-north-west of Cape Crossfire. The rocks are generally altered and have medium-to-large grained textures. The mineral assemblage consists of plagioclase, rare quatz, horneblende, subordinate orthopyroxene and biotite. The opaque minerals content is small, mainly formed by ilmenite and spinel.

No Ridge

The samples were collected at the so-called Marinella Camp locality, that is reported as No Ridge by the Ganovex Geological Map[NN]. A medium-to-large grained monzo-syenite crops out from the inner portion of this area toward the cliff. It consist of large K-feldspar, subordinate quartz adn plagioclase and arfvesonidic honeblende, rare clinopyroxe, and accessory monazite, ilmenite and magnetite. The monzo-syenite likely emplaced in different stages, as geochronological data consistently indicate 32 Ma and 26 Ma, respectively. We used the 31.8 Ma age on the basis of lithotype similarity and areal proximity of the measured samples to the dated one.

Data

Supplementary Table 1. Oxygen isotope data for standards measured in the course of the study. $\Delta'^{17}O$ calculated for λ=0.528.

Sample	$\delta^{17}O$	$\delta^{18}O$	$\delta'^{17}O$	$\delta'^{18}O$	$\Delta'^{17}O$	Iterations
NBS28	5.046	9.630	5.033	9.584	-0.027	30
NBS28	5.093	9.727	5.081	9.680	-0.030	30
NBS28	5.184	9.893	5.171	9.844	-0.027	30
L1std	9.662	18.551	9.616	18.381	-0.090	30
L1std	9.240	17.716	9.198	17.561	-0.075	30
L1std	9.425	18.099	9.381	17.937	-0.090	30
L1std	9.540	18.350	9.495	18.184	-0.106	30
L1std	9.180	17.654	9.138	17.500	-0.102	30
L1std	9.694	18.645	9.647	18.473	-0.107	30
L1std	9.718	18.659	9.671	18.487	-0.090	30
L1std	9.690	18.605	9.643	18.434	-0.090	30
L1std	9.602	18.435	9.556	18.267	-0.089	30
L1std	9.583	18.397	9.537	18.230	-0.088	30
L1std	9.485	18.208	9.440	18.044	-0.087	30
L1std	9.519	18.304	9.474	18.138	-0.103	30
L1std	9.546	18.331	9.501	18.165	-0.090	30
L1std	9.653	18.533	9.607	18.363	-0.089	30
L1std	9.648	18.532	9.602	18.362	-0.094	30
L1std	9.698	18.666	9.651	18.494	-0.114	30
L1std	9.661	18.547	9.615	18.377	-0.088	30
L1std	9.525	18.290	9.480	18.125	-0.090	30
L1std	9.344	17.945	9.301	17.786	-0.090	30
L1std	9.358	17.972	9.314	17.812	-0.090	30
L1std	9.818	18.851	9.770	18.676	-0.091	30
L1std	9.644	18.529	9.598	18.359	-0.096	30
L1std	9.659	18.513	9.613	18.344	-0.073	30
L1std	9.793	18.798	9.745	18.624	-0.088	30
L1std	9.740	18.702	9.693	18.529	-0.091	30
L1std	9.496	18.218	9.451	18.054	-0.081	30
L1std	9.477	18.196	9.432	18.032	-0.089	30
L1std	9.331	17.936	9.288	17.777	-0.099	30
L1std	9.298	17.843	9.255	17.686	-0.083	30
L1std	9.374	17.995	9.330	17.835	-0.087	30
L1std	9.335	17.910	9.292	17.752	-0.081	30
L1std	9.503	18.251	9.458	18.086	-0.092	30
L1std	9.512	18.264	9.467	18.099	-0.089	30

Supplementary Table 2. Oxygen isotope data for Antarctic samples. $\Delta'^{17}O$ calculated for λ=0.528

Site	Sample	Rock type	$\delta^{17}O$	$\delta^{18}O$	$\delta'^{17}O$	$\delta'^{18}O$	$\Delta'^{17}O$	SEM	Rep.	Iter.
Styx Gl.	LR102	Monzo-Syenite	-1.710	-3.126	-1.711	-3.131	-0.058	0.002	2	30
Styx Gl.	LR103	Monzo-Syenite	0.967	1.993	0.967	1.991	-0.085	0.006	2	30
Styx Gl.	CD202	Monzo-Syenite	-0.623	-1.068	-0.623	-1.069	-0.059	0.002	2	30
Styx Gl.	CD203	Monzo-Syenite	-0.117	-0.088	-0.117	-0.088	-0.071	0.003	2	30
Styx Gl.	CD204	Monzo-Syenite	-1.271	-2.288	-1.272	-2.291	-0.062	0.001	2	30
Styx Gl.	SX3	Monzo-Syenite	-1.542	-2.786	-1.543	-2.790	-0.070	N/A	1	30
Styx Gl.	SX4	Monzo-Syenite	-1.171	-2.088	-1.172	-2.090	-0.068	N/A	1	30
Styx Gl.	SX5	Monzo-Syenite	-0.242	-0.368	-0.242	-0.368	-0.048	N/A	1	30
Oakley Gl.	DF1	Monzo-Syenite	2.819	5.441	2.815	5.426	-0.050	N/A	1	30
Oakley Gl.	DF10	Monzo-Syenite	3.068	5.926	3.063	5.909	-0.056	N/A	1	30
Oakley Gl.	DF9	Monzo-Syenite	4.319	8.326	4.310	8.292	-0.068	N/A	1	30
Oakley Gl.	CD47	Monzo-Syenite	0.890	1.829	0.890	1.827	-0.075	0.008	2	30
Oakley Gl.	CD54	Monzo-Syenite	5.657	10.890	5.641	10.831	-0.078	0.002	2	30
Oakley Gl.	CD48	Monzo-Syenite	1.903	3.689	1.901	3.682	-0.044	0.003	2	30
Oakley Gl.	LR71	Monzo-Syenite	4.573	8.806	4.562	8.767	-0.067	0.002	2	30
Oakley Gl.	SAX5	Monzo-Syenite	3.819	7.333	3.812	7.306	-0.046	N/A	1	30
Mt.McGee	LR172	Monzonite	4.510	8.664	4.499	8.626	-0.055	0.004	2	30
Mt.McGee	CD212	Monzonite	4.150	7.983	4.141	7.952	-0.057	N/A	1	30
Mt.McGee	CD318	Monzonite	4.533	8.718	4.523	8.680	-0.060	N/A	1	30
Mt.McGee	CD218	Monzonite	4.116	7.900	4.107	7.869	-0.047	N/A	1	30
Mt.McGee	LR142	Monzonite	3.636	6.989	3.629	6.964	-0.048	0.006	2	30
Mt.McGee	LR93	Monzonite	4.740	9.108	4.729	9.067	-0.058	0.008	2	30
Mt.McGee	LR97	Monzonite	6.221	11.953	6.202	11.882	-0.072	0.002	2	30
Mt.McGee	LR137	Monzonite	4.520	8.677	4.510	8.640	-0.052	0.003	2	30
Mt.McGee	LR151	Monzonite	5.738	11.028	5.721	10.967	-0.070	0.002	2	30
Mt.McGee	LR24b	Monzonite	6.572	12.619	6.550	12.540	-0.071	0.003	2	30
Mt.McGee	CD190	Granite	7.381	14.144	7.354	14.045	-0.062	0.001	2	30
Mt.McGee	CD192	Granite	7.209	13.803	7.183	13.708	-0.055	0.005	2	30
Mt.McGee	CD189	Granite	7.291	13.964	7.265	13.867	-0.057	0.001	2	30
Mt.McGee	LR98	Granite	7.283	13.943	7.257	13.846	-0.054	0.007	2	30
Mt.McGee	LR23	Granite	6.120	11.722	6.101	11.654	-0.052	N/A	1	30
Mt.McGee	LR30	Granite	6.760	12.952	6.737	12.869	-0.058	N/A	1	30
Mt.McGee	LR128	Granite	7.146	13.694	7.121	13.601	-0.061	0.001	2	30
Mt.McGee	CD182qzA	Granite	6.804	13.053	6.781	12.969	-0.066	0.003	2	30
No Ridge	LR75	Monzonite	-1.441	-2.622	-1.442	-2.625	-0.056	0.003	2	30
No Ridge	LR76	Monzonite	-3.649	-6.862	-3.655	-6.886	-0.020	0.004	2	30
No Ridge	LR77	Monzonite	0.057	0.227	0.056	0.226	-0.063	0.008	2	30
No Ridge	LR78	Monzonite	1.603	3.174	1.602	3.169	-0.072	0.004	2	30
No Ridge	D1a	Monzonite	4.245	8.228	4.236	8.194	-0.091	N/A	1	30
No Ridge	D2b	Monzonite	1.384	2.772	1.383	2.768	-0.079	N/A	1	30
Cape King	LR9	qz-Diorite	3.728	7.175	3.721	7.149	-0.054	N/A	1	30
Cape King	CD259	Diorite	4.376	8.414	4.366	8.379	-0.058	N/A	1	30
Cape King	LR8	qz-Diorite	2.038	3.997	2.036	3.989	-0.070	N/A	1	30
Cape King	CD260	Diorite	3.772	7.273	3.765	7.247	-0.061	0.008	2	30
Cape King	LR6	qz-Diorite	3.235	6.268	3.230	6.248	-0.065	0.004	2	30
Cape King	LR7	qz-Diorite	4.229	8.120	4.220	8.087	-0.050	0.002	2	30
Cape Crossfire	AR14	Monzonite	4.211	8.147	4.203	8.114	-0.082	0.003	2	30
Cape Crossfire	AR15	Monzonite	4.083	7.893	4.074	7.862	-0.077	N/A	1	30
Cape Crossfire	AR18	Monzonite	4.171	8.071	4.162	8.039	-0.082	0.002	2	30
Cape Crossfire	AR20	Monzonite	3.462	6.728	3.456	6.705	-0.085	N/A	1	30
Cape Crossfire	D2	Monzonite	1.422	2.871	1.421	2.867	-0.093	N/A	1	30
Cape Crossfire	AR21	Monzonite	4.326	8.385	4.317	8.350	-0.092	N/A	1	30

Supplementary Table 3. Definitions and equations.

The δ^{17}Oand δ^{18}O values are defined as:

$$\delta^{x}O = \left(\frac{R_{sample}}{R_{standard}} - 1\right) \times 1000 \quad (5)$$

where R is the $^{18}O/^{16}O$ or $^{17}O/^{16}O$ ratio, depending on *x*.

The interaction between quartz and hydrothermal waters was modelled using the following mass balance mixing equation (e.g. Taylor, 1977):

$$X * \delta^{n}O^{i}_{qz} + (1 - X) * \delta^{n}O^{i}_{H2O} = X * \delta^{n}O^{f}_{qz} + (1 - X) * \delta^{n}O^{f}_{H2O}\ X \quad (6)$$

where δ^n is the δ^{17}O or δ^{18}O value, the ^{f} and ^{i} indexes are referred to final and initial δ^n –values, respectively, and *X* is the water/mineral ratio, that is also the extent of quartz alteration, from 0 to 100%.

In the quartz–water system at isotopic equilibrium, the $\delta^{18}O_{qz}{}^{f}$ value is determined by isotopic equilibration with the meteoric-hydrothermal water ($\delta^{18}O_{H2O}{}^{i}$) for each water/mineral ratio, thus by the fractionation factor between quartz and water. The latter is expressed as: $\alpha_{qz\text{-}water} = (1000 + \delta_{qz})/(1000 + \delta_{H2O})$ and varies with temperature according to the relation:

$$1000 ln\alpha_{qz-H2O} = \frac{A * 10^6}{T^2} + \frac{B * 10^3}{T} \quad (7)$$

where the parameters A = 4.28, B= 3.5, (Sharp et al., 2016). The fractionation factors for ^{18}O and ^{17}O are can be expressed by the relation:

$$\alpha^{17}O_{A-B} = (\alpha^{18}O_{A-B})^{\theta} \quad (8)$$

In the quartz-water system the value of θ is given by the expression:

$$\theta_{Qz-H2} = -\frac{1.85}{T} + 0.5305 \quad (9)$$

Therefore, the variation of $\alpha^{17}O_{qz-H2}$ with temperature can be written as:

$$1000\ ln\ \alpha^{17}O_{qz-H2O} = \left(\frac{4.28 * 10^6}{T^2} + \frac{3.5 * 10^3}{T}\right)\left(0.5305 - \frac{1.85}{T}\right) \quad (10)$$

Accordingly, the $\delta^n\ O_{qz}{}^{f}$ can be obtained from the mass balance equation reported as:

$$\delta^{n}O^{f}_{qz} = \frac{1000 + \alpha\,(\ X \quad \delta^{n}O^{i}_{qz} - X \quad \delta^{n}O^{i}_{H2O} - \delta^{n}O^{i}_{qz} - 1000\ \ X)}{\alpha X - \alpha - \ X} \quad (11)$$

The calculated $\delta^{n}O^{f}_{qz}$ values can be reported in their linearized version $\delta\ ^{n}O^{f}_{qz}$ (Miller 1991), that is defined as:

$$\delta\ ^{n}O^{f}_{qz} = 1000 \ln\left(\frac{\delta^{n}O^{f}_{qz}}{1000} + 1\right) \quad (12)$$

The $\Delta\ ^{17}O$ value is defined as:

$$\Delta\ ^{17}O_{qz} = \ \delta\ ^{17}O^{f}_{qz} - 0.528\,\delta\ \ ^{18}O^{f}_{qz} \quad (13)$$

Supplementary Information_Model Data

Area	$^{18}O_{water}$	Range $^{18}O_{water}$	$'^{18}O_{water}$	$'^{17}O_{water}$	$'^{17}O_{rock}$	$^{18}O_{rock}$	$'^{18}O_{rock}$	$'^{17}O_{rock}$
South Oakley Glacier	-9.058	± 2	-9.100	0.030	-0.090	12.070	12.000	6.246
Mt. McGee	-14.543	± 2	-14.650	0.030	-0.060	15.010	14.900	7.807
Styx Gl.	-22.004	± 3	-22.250	0.030	-0.085	9.000	8.960	4.640
Cape Crossfire	-28.000	± 3	-28.400	0.030	-0.085	9.040	9.000	4.700
Cape King	-30.039	± 3	-30.500	0.030	-0.052	9.040	9.000	4.700
No Ridge	-19.016	± 1	-19.200	0.030	-0.092	10.050	10.000	5.190

Supplementary Table 4. Hydrogen isotope composition for Antarctic samples.

Abbreviations: bt = biotite; am = amphibole

Mt. McGee		Oakley Gl.		Cape King		No Ridge		Styx Gl.		Cape Crossfire (35)	
**sample**	**dD**	**sample**	**dD**	**sample**	**dD**	**sample**	**dD**	**sample**	**dD**	**sample**	**dD**
LR 137 bt	-83	CD48 am	-118	LR 7 am	-170	LR 78 am	-135	LR103 am	-195	AR14 am	-185
LR 142 bt	-142	DF9 am	-169	LR 8 am	-179	D1a am	-142	CD202 am	-214	AR18 am	-228
LR 151 bt	-115	DF10 am	-112	LR 6 am	-181	D2a am	-185	SX4 am	-177	AR20 am	-177
LR 172 bt	-93	DF3 am	-173	CD 261 bt	-179	LR71 am	-156	SX5 am	-181		
LR 98 bt	-93	DF1 am	-168	L1 am	-187						
LR 97 bt	-89	DF8 am	-216	CD260 bt	-198						
LR 24 bt	-124			CD259 bt	-254						
LR 23 bt	-176										
LR 30 bt	-174										
C8 bt	-183										
C1 bt	-179										
LR 112 am	-191										
LR 137 am	-136										
LR 142 am	-167										
LR 151 am	-145										
LR 128 bt	-175										
LR 157 am	-135										
SX4 am	-177										
CD205 am	-127										
CD318 am	-129										
CD341 am	-116										
CD218 am	-127										

Supplementary Table 5. Estimates of temperature variations based on $\delta^{18}O$/T(°C) rates

		**Cape king**	**Cape Crossfire**	**No Ridge**	**Mt. McGee**	**Oakley Gl.**	**Styx Gl.**
**Age**		**34 Ma**	**35 Ma**	**32 Ma**	**38Ma**	**40Ma**	**35 Ma**
$\delta^{18}O_w$		**-30**	**-28**	**-19**	**-14.5**	**-9**	**-22**
**T °C (S00)**		-26.5	-24.1		-7.4	-0.6	
**T °C (MD08)**		-27.4	-24.9		-8.0	-1.1	
**T °C RAG92**		-26.5	-23.1		-14.5	-9.0	
		**Δ temp 38-34**					
**T °C (S00)**		-19.1					
**T °C (MD08)**		-19.4					
**T °C RAG92**		-12.0					

Supplementary References:

1. GANOVEX Team. Geological map of North Victoria Land, Antarctica, 1: 500 000, Explanatory Notes. Geologisches Jahrbuch, **B66**, 7-80 (1987).
2. Dallai, L., & Burgess, R. A record of Antarctic surface temperature between 25 and 50 million years ago. Geology, **39**, 423–426 (2011).
3. Miller, M.F. Isotopic fractionation and the quantification of ^{17}O anomalies in the oxygen three-isotope system: an appraisal and geochemical significance. Geochim. Cosmochim. Acta, **11**, 1881–1889 (2002).
4. Taylor, H. P. Jr. Water/rock interactions and the origin of H2O in granitic batholiths Journal of the Geol. Soc. Lond. **33**, 509-558 (1977).
5. Sharp, Z. D., Gibbons, J. A., Maltsev, O., Atudorei, V., Pack, A., Sengupta, S., Shock, E. L., Knauth, L. P. A calibration of the triple oxygen isotope fractionation in the SiO2-H2O system and applications to natural samples. Geochim. Cosmochim. Acta **186**, 105–119 (2016).
